# Research in orthopaedic trauma surgery: approaches of basic scientists and clinicians and the relevance of interprofessional research teams

**DOI:** 10.1007/s00068-022-02110-x

**Published:** 2022-09-23

**Authors:** Frank Hildebrand, Christine Höfer, Klemens Horst, Benedikt Friemert, Dietmar Pennig, Ingo Marzi, Richard Stange, Scientific Committee of the German Society for Trauma Surgery

**Affiliations:** 1 Department of Orthopedics, Trauma and Reconstructive Surgery, University Hospital RWTH Aachen, Pauwelsstrasse 30, 52074 Aachen, Germany; 2 AUC-Academy for Trauma Surgery, Munich, Germany; 3 Department of Orthopedics, Trauma, Septic and Reconstructive Surgery, Sports Traumatology, German Army Hospital Ulm, Ulm, Germany; 4 German Society for Trauma Surgery, Berlin, Germany; 5 Department of Trauma, Hand and Reconstructive Surgery, Hospital of the Johann Wolfgang Goethe-University Frankfurt am Main, Frankfurt/Main, Germany; 6 Department of Regenerative Musculoskeletal Medicine, Institute for Musculoskeletal Medicine, University Hospital Münster, Munich, Germany

**Keywords:** Basic scientist, Scientifically active clinician, Research teams, Interdisciplinarity, Interprofessional cooperation

## Abstract

**Background:**

An increasing clinical workload and growing financial, administrative and legal burdens as well as changing demands regarding work-life balance have resulted in an increased emphasis on clinical practice at the expense of research activities by orthopaedic trauma surgeons. This has led to an overall decrease in the number of scientifically active clinicians in orthopaedic trauma surgery, which represents a serious burden on research in this field. In order to guarantee that the clinical relevance of this discipline is also mirrored in the scientific field, new concepts are needed to keep clinicians involved in research.

**Methods:**

Literature review and discussion of the results of a survey.

**Results/conclusion:**

An interdisciplinary and -professional team approach involving clinicians and basic scientists with different fields of expertise appears to be a promising method. Although differences regarding motivation, research focuses, funding rates and sources as well as inhibitory factors for research activities between basic scientists and clinicians exist, successful and long-lasting collaborations have already proven fruitful. For further implementation of the team approach, diverse prerequisites are necessary. Among those measures, institutions (e.g. societies, universities etc.) must shift the focus of their support mechanisms from independent scientist models to research team performances.

## Introduction

The sophisticated pathophysiology of musculoskeletal diseases and injuries is associated with an increasing complexity of clinical and experimental approaches toward understanding the underlying mechanisms. Knowledge about these processes is imperative for further optimization of diagnostic and therapeutic approaches. Interestingly, strong scientific engagement of institutions has also been associated with improved treatment results, indicating that research involvement might also beneficially affect patient care [1].

However, despite the advances made in understanding disease mechanisms, these do not necessarily result in new treatments, diagnostics and prevention. For successful transfer from bench to bedside, the role of scientifically active clinicians as a nexus between basic science and clinical practice cannot be overstated. In this context, this group of clinicians is of the utmost importance for the translation of study results from basic science into the clinic, the transfer of clinically relevant research questions to the lab and the conduct of clinical research and trials [2–6]. Despite their relevance to scientific progress, scientifically active clinicians face numerous financial, administrative and legal burdens, which are difficult to handle in addition to the high clinical workload [3, 5]. This is especially true for surgical disciplines with a high number of emergency cases and severe restrictions imposed by the time schedule of the operating room, such as (orthopaedic) trauma surgeons. The number of scientifically active clinicians has decreased in many countries, including both young clinicians entering their research careers and experienced clinician researchers not being able to sustain their engagement [1, 3–5, 7]. As a result, funding rates in the field of (orthopaedic) trauma surgery also do not reflect the relevance of this discipline within the medical field. Basic scientists, who perform the majority of biomedical research, also face significant challenges. However, these are likely to differ, at least partly, from those of clinicians.

In order to increase the scientific impact of (orthopaedic) trauma surgery, there is an urgent need to establish comprehensive research structures. Among diverse measures, structured promotion of scientifically active clinicians (clinicians/physician scientists) and support for the establishment of interdisciplinary team structures including clinicians and basic scientists have a particular potential to facilitate knowledge transfer in (orthopaedic) trauma surgery.

## Different perspectives of scientifically active clinicians (physician/clinician scientists) and basic scientists—results of a national survey

Although scientifically active clinicians and basic scientists often share the same type of scientific and mechanistic ideas [2], it is essential to understand the individual research focuses, perspectives and expectations of these groups in order to promote common research activities. Therefore, we investigated these factors for scientifically active clinicians and basic scientists in a sub-analysis of a national survey of the Scientific Committee of the German Society for Trauma Surgery. The questionnaire included 11 questions, particularly focusing on current and future research focuses, financing of the current research projects and factors inhibiting the performance of research. Further details of the survey can be found in a previous publication by our group [8].

**Fig. 1:**
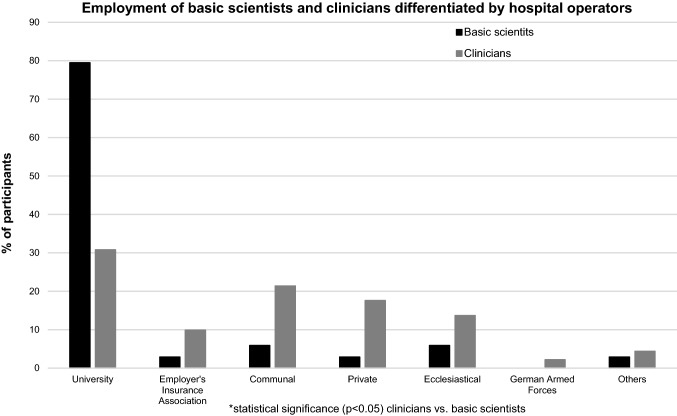
Employment of basic scientists and clinicians differentiated by hospital operator

Among the 229 participants, 190 were clinicians (83%). Of the basic scientists, 87.0% worked in a level I centre (level II: 6.5%, level III: 6.5%), whereas only 51.7% of the participating clinicians were employed at a level I centre (level II: 22.8%, level III: 25.5%). Thereby, a positive correlation between basic scientists and employment at a level I centre was found (*r* = 0.204, *p* < 0.001). The hospital operators for basic scientists and clinicians are presented in Fig. 1. Here, work as a basic scientist was positively correlated to employment at a university hospital (*r* = 0.305, *p* < 0.001).

**Fig. 2:**
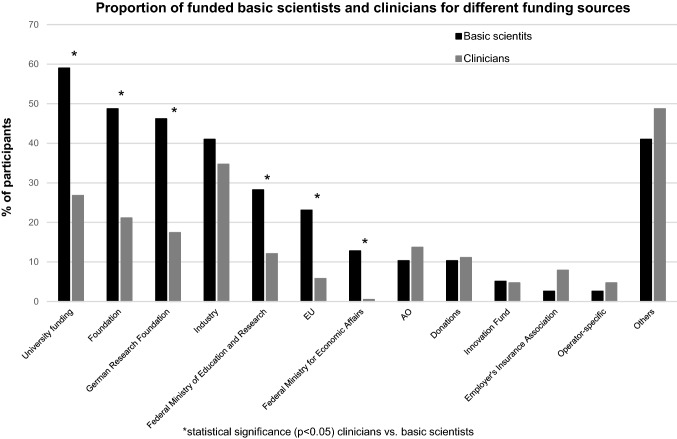
Proportion of funded basic scientists and clinicians for different funding sources, *statistical significance (*p* < 0.05)

Focusing on research funding, a significantly higher proportion of basic scientists were sponsored by foundations as well as university and public (German research council, EU, federal ministries) funding. Industrial sponsorship was comparable between basic scientists and clinicians (Fig. 2). About two thirds (65.7%) of basic scientists but only 28% of clinicians had current funded cooperative or consortium projects. This resulted in a positive correlation (*r* = 0.296, *p* < 0.001) between funding and working as a basic scientist.

**Fig. 3:**
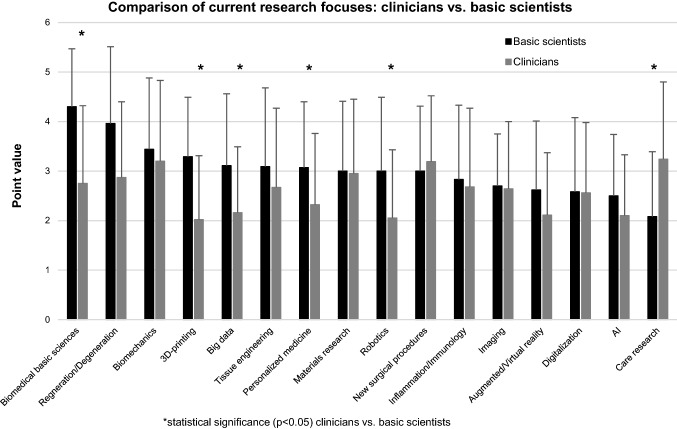
Comparison of current research focuses between clinicians and basic scientists based on assigned point values (0 = no relevance to 5 = highest relevance), * statistical significance (*p* < 0.05)

Basic scientists assigned the highest relevance of their current research focuses to ‘Biomedical basic sciences’. The importance of this topic was significantly higher for basic scientists than for clinicians. Compared with clinicians, basic scientists assigned greater importance to subjects in the field of ‘New technologies’. By contrast, ‘Care research’ was significantly more relevant to clinicians than to basic scientists. Also, the field of ‘New surgical procedures’ tended to be of greater significance to clinicians than to basic scientists (Fig. 3).

**Fig. 4:**
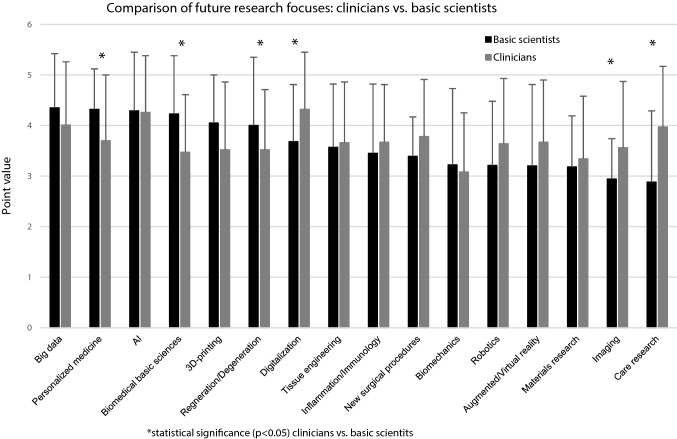
Comparison of future research focuses between clinicians and basic scientists based on assigned point values (0 = no relevance to 5 = highest relevance), *statistical significance (*p* < 0.05)

**Fig. 5:**
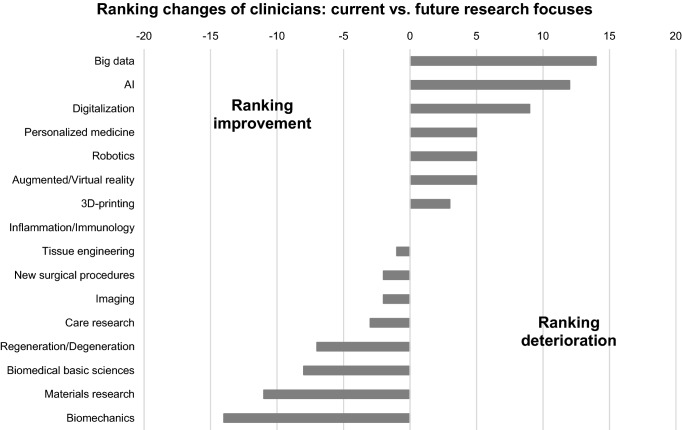
Relevant changes of different research focuses between the current and the future situation of clinicians

**Fig. 6:**
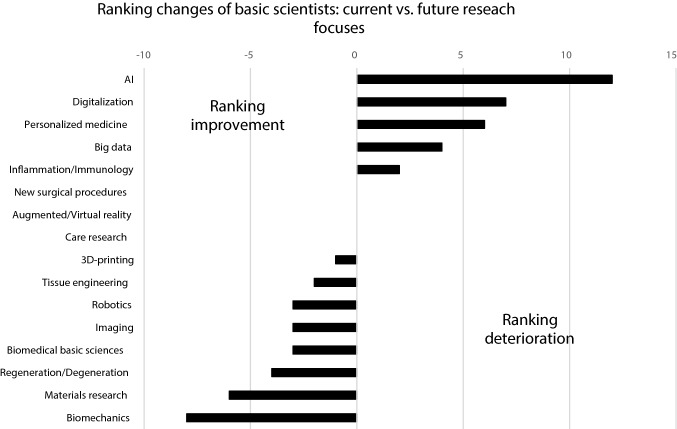
Relevant changes of different research focuses between the current and future situation of basic scientists

The aforementioned differences between clinicians and basic scientists regarding the subjects ‘Biomedical basic sciences’, ‘Care research’ and ‘New surgical procedures’ are also expected for future research focuses. In general, the future relevance of all topics in the field of ‘New technologies’ is expected to increase for clinicians, resulting in a significantly higher importance of ‘Digitalization’ and ‘Imaging’ for clinicians compared to basic scientists (Fig. 4). Basic scientists also assume an enhanced relevance of different aspects in the field of ‘Digital Health’ (‘AI’, ‘Digitalization’, ‘Personalized Medicine’, ‘Big Data’), whereas others are suggested to maintain (‘Augmented/Virtual reality’) or lose (‘Robotics’) their significance. Among the traditional research focuses, ‘Inflammation/Immunology’ is the only topic that is predicted to maintain (clinicians) or to increase (basic scientists) its relevance (Figs. 5 and 6).

**Fig. 7:**
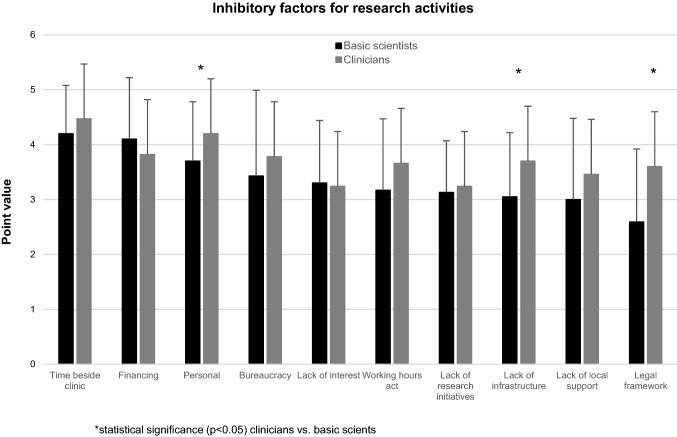
Comparison of factors inhibitory to research between clinicians and basic scientists based on assigned point values (0 = no relevance to 5 = highest relevance), *statistical significance (*p* < 0.05)

Staff shortages and a lack of time outside of clinical work represented the most relevant factors that prevented clinicians from conducting research. In general, clinicians perceived the barriers to research as more severe than basic scientists did, likely reflecting the dual challenge of science and patient care. In this context, significant differences were observed for the aspects ‘Personal’, ‘Lack of infrastructure’ and ‘Legal framework’. Only the topic ‘Financing’ tended to represent a problem that affected basic scientists more than clinicians (Fig. 7).

## Motivation to perform research

How to create or preserve motivation and incentives for scientifically interested or already-active clinicians to perform research currently remains an intensely debated issue [7, 9–11]. In this context, diverse studies have reported that particularly in young scientifically active clinicians, factors such as positive outcome expectations and increased self-efficacy are important prerequisites of conducting research. These aspects are particularly enhanced if personally defined aims are aligned with externally valued success. To maintain research efforts, early research success with sufficient mentorship and adequate training programs, including the scientific development from smaller studies to larger research projects, is a major motivator. In addition, training and mentorship build the confidence to be efficacious in research and to disregard setbacks or barriers [1, 10, 12–14]*.* In more experienced clinicians, Cianciolo et al. [12] also identified a lack of professional identification as a researcher or scientist. This has negative effects on the continuation of research efforts in their later careers. Independently of the stage of their careers, clinicians primarily define research success by its clinical impact (bench to bedside approach) and not primarily by citations and impact factors [12]. Therefore, they preferably perform studies intended to address the demands of their clinical work [5, 15, 16]*.*

By contrast, basic scientists have decided explicitly to pursue a professional scientific career in a university environment and are motivated by bench research with involvement in grant proposals, publishing articles and the performance of experiments. They are less focused than clinicians on early success. In this context, basic scientists are prepared to maintain their research activities even when unfavourable events, such as drawbacks during the experiments or an unintentional transition from one research project to another, occur [12]. However, this group also differentiates between projects that are significant for the professional career and work that is important for the individual. Therefore, both aspects must be considered to promote personal and professional satisfaction [12].

The aforementioned contexts are potentially mirrored by the current and future research focuses of basic scientists and clinicians, which were mentioned in the survey presented here (Figs. 3 and 4). With respect to the current research focuses, ‘new technologies’ such as Big Data, 3D printing, Personalized Medicine and robotics were significantly more important for basic scientists than for clinicians (Fig. 3). Due to the fact that many of these technologies are so far not implemented in the clinical routine of most hospitals and as the research success of clinicians is associated with the clinical impact, this might explain why these subjects are not yet considered of high scientific importance. This assumption is supported by the fact, that ‘care research’, a field with an already high clinical impact, has a significantly higher relevance as a current research focus for clinicians than for basic scientists. For the future, it is common sense that the clinical importance of the ‘new technologies’ will increase significantly [17–19]. Accordingly, their role as future research focuses of clinicians is also expected to increase in the presented survey (Figs. 4 and 5). Beside all mentioned differences between basic scientists and clinicians, it seems worth noting that AI, Big Data, Personalized Medicine and Digitalization are the four topics expected to gain the greatest importance for both groups (Figs. 5 and 6).

Also, for the recruitment of scientific offspring, it is of the utmost importance to motivate students to perform research. Recently, basic science and other research topics have not played a relevant role in medical school curricula despite the steady increase in scientific knowledge. By enabling more effective use of new scientific information, it has been well described that consideration of research topics within the curricula is associated with improved clinical skills. Therefore, the focus of modern medical education should be placed on the acquisition, interpretation and application of new scientific data that also includes the reliable assessment of the effects on outcomes [20–23]. Furthermore, medical students must be involved early in research and scholarly activities (hands-on experience) to arouse their interest in scientific work, at best with participation in the study design. Thereby, the curiosity of the students is promoted, and the likelihood of the application of the acquired skills to further research questions or clinical practice is significantly increased. By contrast, students with no early exposure to scientific questions might be more likely to perceive barriers to performing research [1].

## Factors inhibitory to performing research

Not only the generation of motivation but also the degradation of inhibitory factors is of the utmost importance in fostering research activities. According to the results of the survey presented here, barriers to conducting scientific research are in general more important for clinicians than for basic scientists (Fig. 7). The inhibitory factors are multifactorial, and many of the findings of the survey are in line with results from the international literature [1, 11].

Here it is uniformly described that the priority of universities and other health care providers is to sustain financial health. Therefore, clinical productivity is expected to increase, thereby taking precedence over research activities. Accordingly, aside from clinical work, time was the most important inhibitory factor in the survey presented here. In this context, it is also important to note that scientific activities (writing grants, performing experiments, writing papers etc.), which are associated with absence from the clinic, might even exert negative effects on the clinical career. Furthermore, research time competes with the needs of an adequate work/home life balance, potentially reducing the available time for scientific activities apart from clinical work [1, 7, 9–11]*.*

At the same time, inadequate personal resources impair the capacity for research [24]. This appears to affect clinicians significantly more than basic scientists (Fig. 7) and not only impedes the capability of experienced clinically active scientists to develop and conduct studies but also weakens their role as mentors for younger clinicians (e.g. pre-submission review of grant applications). Thereby, young clinicians are particularly affected by the lack of both role models and time due to clinical obligations. As this might already hamper their start of a potential research career, it is likely that these persons will also have difficulties finding their way into the scientific community at a later time point in their careers. Although some research-compounding factors affect all medical disciplines (e.g. a sense of responsibility to the patient and the ambition for a clinical career), they are particularly emphasized in surgical fields such as (orthopaedic) trauma surgery for different reasons. Among others, the incidence of emergency cases is very high, which causes scheduling difficulties. Furthermore, long periods in the operating room during regular working hours and many on-calls make communication with scientific partners difficult. Also, (orthopaedic) trauma surgeon-specific character traits (e.g. low frustration tolerance, impatience) must be considered, as they result in immediate expectations of success [24]. This stands in contrast to the need for persistence in scientific work and grant submissions, which requires training.

The aforementioned staff shortage and clinical obligations might also be the reason for which clinicians in our survey perceived administrative and legal challenges (e.g. documentation, data protection, working hour act) as a significantly greater hinderance than basic scientists (Fig. 7). It is without question that the aforementioned factors are also major barriers for basic scientists [24]; however, it seems to be easier for them to handle such barriers, e.g. due to fewer time constraints during regular working hours. In this context, it seems worth noting that legal aspects had the lowest inhibitory effects on basic scientists in our survey. Another closely associated problem is that administrative support from institutions is often insufficient to compensate for the steadily deteriorating formal paper work [24]. Accordingly, in our survey, a lack of local support was also a relevant factor inhibiting the research activities of clinicians, whereas basic scientists perceived this aspect as one of the least influential barriers to performing research (Fig. 7).

The same is true for the lack of research infrastructure. Accordingly, previous studies reported that a lack of infrastructure at the department level (e.g. insufficient space for research activities) contributes to an inadequate pool of scientifically active clinicians in (orthopaedic) trauma surgery [1, 24]. Furthermore, it has been well described that the optimal scientific infrastructure for training basic scientists is much better defined than that for scientifically active clinicians and that the relatively few specialized clinical research training programs often have limited capacities at many institutions [2].

The aforementioned different perceptions of the impeding effects of both, infrastructure and local support might be at least partly explained by their significantly higher success rate in generating research funding. In this context, compared to clinicians, the proportion of funded basic scientists was significantly higher for almost all funding sources. This was especially true for intramural funds and foundations but was also true for all public sources (e.g. German Research Foundation, Federal ministries, EU) (Fig. 2). With grant-associated financial resources, basic scientists are able to better cover the increasing costs of research, which include infrastructural aspects such as equipment and space. At the same time, funding promotes the allocation of limited laboratory space at many institutions, so basic scientists have an additional advantage here. Better funding is also associated with improved personnel resources that in turn help to handle administrative and legal aspects.

The higher funding rates for basic scientists are partly explained by several factors. First, funding priorities frequently do not favour studies with a direct clinical impact. Second, grants are often awarded based on a history of publications in top journals rather than on the contribution of study results to the advancement of medicine [6]. Thereby, scientifically active clinicians are potentially disadvantaged. As rejection of research proposals represents the most important reason for feeling unsuccessful and has been identified as a major barrier to scientific engagement [1, 12], this might additionally discourage clinicians from continuing research activities, due to the discrepancy between their engagement and external appreciation and support. As compensation, many scientifically active clinicians seem to perform smaller research projects to influence clinical practice and to achieve recognition. The aforementioned aspects are even aggravated by a general decrease in research funding in the context of increasing the costs of conducting it [24].

Besides the abovementioned medical, administrative, legal and financial factors that make sustained research efforts difficult [4, 5], family and lifestyle issues also have a relevant impact on research activities. This is true for basic scientists but is especially true for scientifically active clinicians, who often have to decide whether to spend their time outside the clinic on research engagements or private obligations.

In conclusion, in an ensuing vicious cycle, the addressed inhibitory factors might result in a further decrease in research activity, particularly in surgical fields such as (orthopaedic) trauma surgery. This in turn results in an expansion of the strategical focus of the universities/institutions to support research in the strongest departments (e.g. conservative medical disciplines) in order to achieve the greatest return on investment (e.g. third-party funding). Thereby, the development of surgical research particularly by clinicians is further disadvantaged. To support surgeons in receiving funding and performing studies, institutions must understand the prerequisites and infrastructure required by this specific group of physicians that is regularly heavily involved in the care of emergency patients and severely restricted by the time schedule of the operating room. Furthermore, institutional expectations for scientific productivity must be adapted to the primary identification of the surgeon as a provider of patient care—even in university hospitals*.*

Attention must also be paid to the fact that the complexity of scientific methods is steadily increasing. This calls for adequate research resources, which can only be provided by a full-time scientist leading a laboratory and representing a contact person for young researchers and clinicians, but not by a surgical leisure time scientist. Furthermore, the importance of statistics, data management and analysis is steadily increasing, which will require specialized knowledge, adequate time to learn them and increasing financial means. This also makes the isolated researcher (basic scientist or clinician) an outdated model, as a single person normally does not possess the necessary diverse skills [2, 24]. Therefore, an interdisciplinary and -professional collaboration between basic scientists and clinicians is imperative if surgeons are to remain involved in the research environment of the institution.

## Potential solutions to foster research: interdisciplinary and -professional teams

Both motivation and the self-perceived relevance of inhibitory factors are influenced by the attitudes of clinicians towards research. In this context, three types were identified: Those with a ‘very positive attitude towards research’, who want to perform research regardless of difficulties and barriers, were differentiated from clinicians with a ‘positive attitude’, who are active in science because they consider it useful but not personally relevant. The third group of ‘unmotivated’ persons does not see the value in research at all [1]. Based on this classification, it is of the utmost importance to maintain the high enthusiasm of the first group and also to awaken and support the personal motivation of the second group, as this is a significant prerequisite for a long-lasting research interest. If possible, unmotivated persons should also be convinced of the fascination of scientific work.

Based on the previous remarks and the findings of the literature, it becomes fairly clear what is needed to foster research activities in (orthopaedic) trauma surgery and to increase the number and diversity of researchers in this field. Besides individual enthusiasm, commitment and motivation, it is important to support young scientifically interested clinicians with mentors, collaborators, accessible infrastructure and structured institutional (funding) support in the very early phase of their careers [1, 13, 25, 26]. For more experienced clinicians, it was suggested that measures to protect research time might be a possibility to keep up the professional identity of clinicians as researchers, thereby improving their motivation to also work as scientists over the long term [5, 16, 27, 28].

There is clear evidence from diverse studies and daily life experience that all these aforementioned steps to positively influence research activities in (orthopaedic) trauma surgery will be more easily achieved with a team approach [1, 6, 24]. In this context, the composition of research teams with clinicians and basic scientists of different disciplines with diverse skills and knowledge, supplemented, if necessary, with epidemiologists and statisticians, is a widely recognized and promising tool to support scientific advance and the translation of scientific findings into clinical practice (cross-disciplinary science) [2, 11, 29]**.** Furthermore, it can be expected that an inspiring and multidisciplinary team will stimulate young clinicians to start scientific projects and experienced clinicians to maintain their research activities as well as to attract external basic scientists to join the institution [2, 24].

The unique role of the (orthopaedic) trauma surgeon within this team is to identify and address clinical problems as well as to lead research into the clinically most relevant and interesting future research directions. Thereby, the performance of research that is better integrated into patient care might be facilitated. By achieving a critical mass, a scientifically competitive research group would provide all competences needed to reliably provide an excellent portfolio of scientific methods as well as epidemiologic and statistical procedures, which are imperatively needed to design, perform and interpret increasingly complex and translational studies in the future. Thereby, relevant prerequisites for ongoing sustainable funding for scientific research projects will also be met. Furthermore, by an interdisciplinary approach, new scientific ideas can be discussed and possibly addressed immediately.

Team members in a multidisciplinary cooperation can also enhance each other’s work in different ways. Scientifically active clinicians without access to their own laboratory have the possibility of collaborating with a basic scientist to investigate a (clinical) research question, while basic scientists may profit by finding easier ways to get in contact with clinicians in order to, for example, match their current and future scientific activities with clinical needs [29]. Sampling of probes from well-characterized patients in cohort or clinical studies might represent a very good example for a potentially successful cooperation between clinicians and basic scientists [2]. The mutual use of existing resources and experimental models is an additional advantage of the team approach.

## Factors in success

The success of the team approach is influenced by different contextual factors. To overcome a potential disruption of interactions between scientifically active clinicians and basic scientists, there are several prerequisites and challenges for a long-lasting and successful cooperation. First, communication between basic scientists and clinicians should be improved [6] and certainly provides the basis for a solid collaboration. Second, studies were traditionally performed by single persons as principal investigators. Therefore, in a team approach, each individual must learn and accept their role as a team member, develop a profound understanding of the strengths of each discipline or profession and feel ownership of the common research questions. Furthermore, a willingness to learn from each other is needed. Therewith, a good and ongoing performance of each team member is likely. Third, new institutional mechanisms to appreciate scientific performances as a team are required. Current research programs are often focused on independent scientist models (e.g. individual training, personalized awards) which do not adequately consider the relevance of the team approach and hinders the acceptance of collaborations. Therefore, inter-disciplinary and -professional training programs must be increasingly implemented and promoted. Fourth, as collaborations often demand more time and resources than individual projects, clinicians need protected time devoted to research activities (learning techniques, meetings and travel time) that is free of clinical responsibilities [1]. This must be ensured by the (orthopaedic) trauma department leaders which have to value the critical importance of scientifically active clinicians in (orthopaedic) trauma. Fifth, the spatial distance between cooperating basic scientists and clinicians must be as low as possible to ensure spontaneous and uncomplicated communication. This colocalization is obviously a critical aspect in translational and interdisciplinary research.

However, there might also be some pitfalls within collaborations. Due to the high clinical burden and the increasing complexity of scientific methods as well as diverse legal and administrative burdens, orthopaedic clinician-scientists in particular may need more help from basic scientists than they can give due to their own obligations, in such things as funding acquisition. On the other hand, basic research personnel is often temporary employed, closely related to distinct research projects. As institutional expectations in terms of funding rates of basic scientists are especially high, this might lead to limited mentoring capacities of basic scientists for young clinicians. Furthermore, clinically orientated research might be difficult to get published in top journals. These issues must be considered by the institutions, so that basic scientists acting as mentors do not experience disadvantages. Furthermore, there might be intense discussions about the ‘relevance’ of participation in research activities (authorship, role in future research grants etc.). This must be clarified at the very beginning of any common research project.

## Conclusion

Without scientifically active clinicians and surgeons, biomedical research disconnects from the process of clinical application. Due to the importance of this, the recruitment and retention of these clinicians represents a critical challenge to the (orthopaedic) trauma community**.** A team approach with clinicians and basic scientists of different fields represents a promising tool to promote the research activities of scientifically interested clinicians. As the results of the survey presented here demonstrate that there are—besides diverse unifying aspects—also some relevant differences regarding funding, research focuses and factors inhibitory to research activities, it is important to bring the souls of basic scientists and clinicians together and to create a win–win situation by identifying the most important criteria for the individual success of each involved person.
